# Interactions of brain, blood, and CSF: a novel mathematical model of cerebral edema

**DOI:** 10.1186/s12987-021-00274-z

**Published:** 2021-09-16

**Authors:** Omer Doron, Yuliya Zadka, Ofer Barnea, Guy Rosenthal

**Affiliations:** 1 Department of Neurosurgery, Hadassah-Hebrew University Medical Center, Kiryat Hadassah, 91120 Jerusalem, Israel; 2 Department of Biomedical Engineering, Tel Aviv University, Tel Aviv, Israel

**Keywords:** ICP, Cerebral edema, Cerebral interstitial fluid, Bulk flow, CSF, Outflow resistance, Lumped parameter model, Blood–brain barrier

## Abstract

**Background:**

Previous models of intracranial pressure (ICP) dynamics have not included flow of cerebral interstitial fluid (ISF) and changes in resistance to its flow when brain swelling occurs. We sought to develop a mathematical model that incorporates resistance to the bulk flow of cerebral ISF to better simulate the physiological changes that occur in pathologies in which brain swelling predominates and to assess the model’s ability to depict changes in cerebral physiology associated with cerebral edema.

**Methods:**

We developed a lumped parameter model which includes a representation of cerebral ISF flow within brain tissue and its interactions with CSF flow and cerebral blood flow (CBF). The model is based on an electrical analog circuit with four intracranial compartments: the (1) subarachnoid space, (2) brain, (3) ventricles, (4) cerebral vasculature and the extracranial spinal thecal sac. We determined changes in pressure and volume within cerebral compartments at steady-state and simulated physiological perturbations including rapid injection of fluid into the intracranial space, hyperventilation, and hypoventilation. We simulated changes in resistance to flow or absorption of CSF and cerebral ISF to model hydrocephalus, cerebral edema, and to simulate disruption of the blood–brain barrier (BBB).

**Results:**

The model accurately replicates well-accepted features of intracranial physiology including the exponential-like pressure–volume curve with rapid fluid injection, increased ICP pulse pressure with rising ICP, hydrocephalus resulting from increased resistance to CSF outflow, and changes associated with hyperventilation and hypoventilation. Importantly, modeling cerebral edema with increased resistance to cerebral ISF flow mimics key features of brain swelling including elevated ICP, increased brain volume, markedly reduced ventricular volume, and a contracted subarachnoid space. Similarly, a decreased resistance to flow of fluid across the BBB leads to an exponential-like rise in ICP and ventricular collapse.

**Conclusions:**

The model accurately depicts the complex interactions that occur between pressure, volume, and resistances to flow in the different intracranial compartments under specific pathophysiological conditions. In modelling resistance to bulk flow of cerebral ISF, it may serve as a platform for improved modelling of cerebral edema and blood–brain barrier disruption that occur following brain injury.

## Introduction

Intracranial pressure (ICP) is the cornerstone of most treatment decisions in the neurointensive care unit. Monro and Kellie presented the hypothesis linking volume and pressure in the closed cranium that served as the basis for our understanding of raised ICP [1, 2]. The physiologist Hugh Davson described steady-state ICP in terms of the flow of cerebrospinal fluid, the resistance to its outflow from the cranium, and the pressure in the sagittal sinus where CSF is absorbed [3, 4]. This equation, which defines steady-state ICP under certain conditions laid the cornerstone upon which all further models of intracranial physiology were built. Davson’s simple, one-element model of CSF hydrodynamics accounts for the value of steady-state ICP when the primary pathology is a disturbance in the ability to reabsorb CSF but does not account for other pathologies that may lead to increased ICP. The pioneering work of Marmarou added the element of intracranial compliance in order to better describe the dynamic aspects of ICP and the pressure changes that occur with injection of different volumes into the closed cranium at varying rates [5]. Further investigators used these models to describe the importance of the rate of volume changes in the cranium on intracranial pressure dynamics [6, 7]. Others developed models to depict obstruction to the flow of CSF within the ventricular system and replicate various clinical aspects of obstructive hydrocephalus [8, 9]. Subsequently, Ursino and others added the important element of the cerebrovascular circulation to models of intracranial fluid dynamics to account for cerebral blood flow (CBF) and its key aspects such as cerebrovascular autoregulation [10–17]. Recent studies have also modeled the ICP waveform and translated an electrical analogue model into a working physical model of the cerebral vascular circulation [18, 19]. Together these models of intracranial hydrodynamics built our current understanding of ICP, CBF, and their inter-relationships and served as the foundation for a vast body of literature describing important interactions between cerebral and systemic physiological parameters [20, 21].

While models of intracranial physiology to date have provided the basis of our understanding of ICP, it is important to point out that fundamentally they were based on two primary elements: the circulation of CSF and the cerebrovascular circulation. As such, they did not take into account a crucial aspect of intracranial physiology, swelling of the brain itself and the effect of raised ICP on the circulation of cerebral interstitial fluid (ISF). Cerebral swelling is a key aspect of many pathologies commonly treated in the neurointensive care unit, including traumatic brain injury (TBI) and stroke. The ability to model the effects of cerebral swelling on intracranial physiology is an important goal. In this work, we aimed to build on the previous classical models of intracranial fluid dynamics adding the additional component of “brain” to those models that have described the flow of “CSF” and “blood.” As such, we sought to develop a model of intracranial hydrodynamics that incorporates the flow of cerebral interstitial fluid. We sought to assess the ability of this model to predict clinically relevant changes in cerebral physiology that are known to occur with cerebral edema and raised ICP.

## Methods

**Fig. 1:**
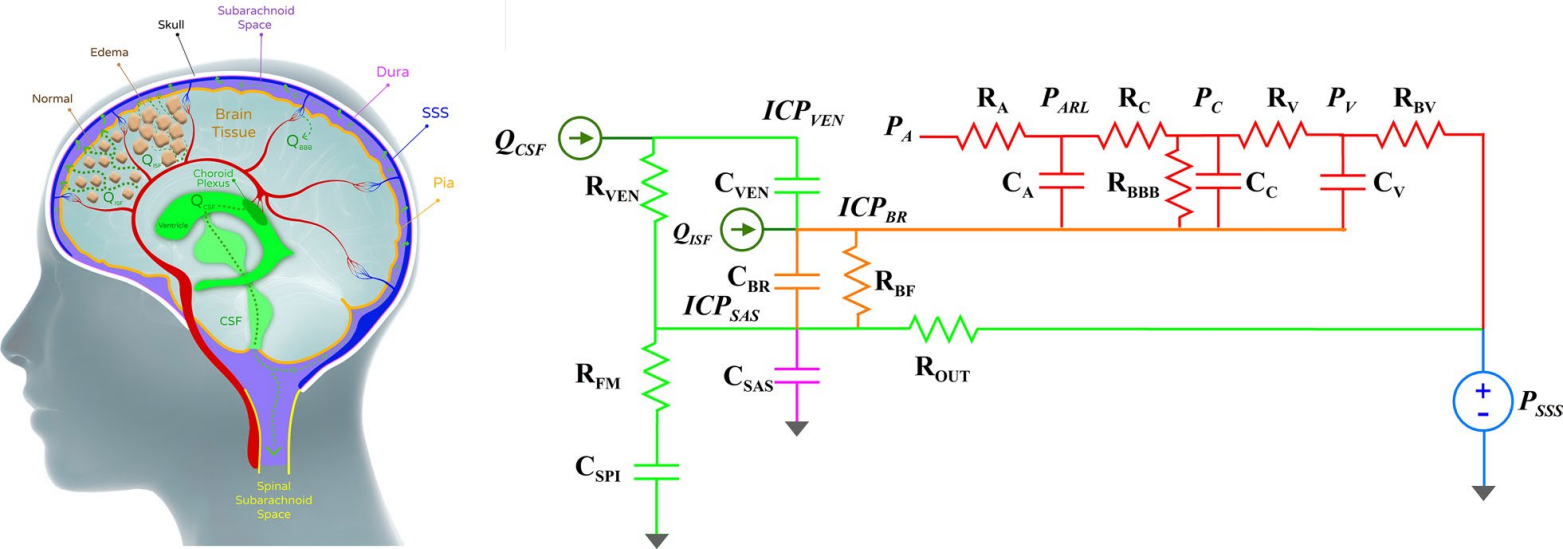
**A** Structural model of the intracranial space with four compartments: the subarachnoid space enveloped inwardly by the pia and outwardly by the dura, the brain enveloped by the pia, the ventricles, and the cerebrovascular circulation. The intracranial subarachnoid space is in continuity with the spinal subarachnoid space. CSF is produced by the choroid plexus (Q_CSF_) and flows through the ventricles and into the subarachnoid space until its absorption into the superior sagittal sinus (SSS) via the arachnoid granulations. The cerebral interstitial fluid (Q_ISF_) flows through the extracellular space and into the subarachnoid space where it joins CSF flow. Cerebral intracellular edema causes a reduction of the extracellular space volume with a concomitant increase in resistance to the flow of cerebral interstitial fluid. When blood–brain barrier (BBB) disruption associated with brain injury occurs, fluid may enter the brain from the capillaries through the disrupted BBB (Q_BBB_) leading to cerebral edema. **B** An electrical analogue model demonstrating flow, resistances, and compliances, within the compartments depicted in the structural model. Flow of cerebral ISF (Q_ISF_) and CSF (Q_CSF_) is depicted in green, while the cerebral vascular circulation is depicted in red. The intracranial compartments are represented by the capacitors C_BR_ (orange), C_VEN_ (green), and C_SAS_ (purple) for brain, ventricles, and subarachnoid space, respectively. The cerebrovascular compartment is represented by the capacitors (red) C_A_, C_C_, and C_V_, representing the arterial-arteriolar, capillary and venous compartments, respectively. The spinal subarachnoid space is represented by C_SPI_. Resistance to bulk flow of cerebral interstitial fluid is represented by R_BF_, resistance to CSF flow in the ventricles by R_VEN_, resistance to CSF outflow by absorption into the superior sagittal sinus (blue) is represented by R_OUT_. Resistance to flow of CSF from the intracranial subarachnoid space through the foramen magnum into the spinal subarachnoid space is represented as R_FM_. Resistance to flow in the cerebrovascular system is represented by R_A_, R_C_, R_V_, and R_BV_, which denote resistance of arterioles, capillaries, veins, and bridging veins, respectively. Resistance to movement of fluid across the BBB, which is very high under normal conditions but may be impaired with brain injury is represented by R_BBB_. Pressure in the intracranial compartments include ventricular pressure, (ICP_VEN_), brain intraparenchymal pressure (ICP_BR_), and subarachnoid space pressure (ICP_SAS_), while those in the cerebrovascular compartment include systemic arterial pressure (P_A_), arteriolar pressure (P_ARL_), capillary pressure (P_C_), and venous pressure (P_V_). Pressure in the superior sagittal sinus is depicted by P_SSS_

We developed a lumped parameter model to represent the interactions between brain tissue, CSF, and blood enclosed within the rigid cranium under both normal physiological conditions and under conditions of brain edema. As in the classical formulations of intracranial physiology, we describe the model using a mathematical formulation that is based on the physical relations in the system and included a representation of these relations using an electrical analog circuit. Figure 1 shows the intracranial anatomy and the corresponding electrical analog model. The model consists of four major compartments and flow conduits. Each compartment contains fluid within an elastic barrier that is represented as an elastic chamber with nonlinear pressure–volume relations. These nonlinear relations may take a form of either an exponent or a polynomial describing the chamber’s transmural pressure as a function of total volume enclosed within that chamber. The selection of exponential or polynomial functions were chosen to accord with accepted intracranial physiological relationships and are detailed below. Importantly, the enclosed volume in some chambers may include the volumes of other elastic chambers that are contained within it. From the outside-in these chambers are: (1) The subarachnoid space that is limited outwardly by the semi-elastic dura and the rigid skull and inwardly by the pia mater but encloses all intracranial volumes including the brain tissue, ISF, CSF, and the cerebrovascular circulation. (2) The brain compartment through which the cerebral interstitial fluid circulates and is limited outwardly by the pia. (3) The ventricles where CSF is formed and through which it flows, and (4) the cerebral vascular tree. The superior sagittal sinus, owing to the thick dural walls of the sinus, is a separate compartment within the cranium. It is the outflow for venous blood and the site of reabsorption for CSF. It maintains a pressure lower than the ICP under most physiological conditions [22–27]. In addition, in the model the spinal thecal sac compartment is interconnected to the intracranial space through the link between the cranial and spinal subarachnoid spaces.

The outermost compartment in the model, the subarachnoid space, is represented as nonlinear elastic chamber in the electrical analog circuit by a capacitor with a nonlinear pressure–volume relationship in the form of an exponential function (Eq. 1):

1 $${ICP}_{SAS}={a}_{s}{e}^{{b}_{s}({V}_{tot}-{V}_{0})}+{c}_{s}$$

${ICP}_{SAS}$ is the subarachnoid pressure, ${V}_{tot}$ is the total cranial volume, which is the sum of the brain compartment volume, ventricular volume, subarachnoid space volume, and blood volume, and ${V}_{0}$ is the cranial unstressed volume. Coefficient values ${a}_{s}$, ${b}_{s}$, and ${c}_{s}$ as well as all the coefficients for the equations listed below are detailed in Appendix. As the capacity of the intracranial space to accommodate additional volume is limited, this equation accounts for the exponential-like rise in ICP when the total volume increases. Previous models have also included a similar equation to account for the exponential-like rise in pressure when intracranial volume reserve capacity reaches its limit [28, 29].

The brain compartment in turn encloses two additional compartments that are, for the most part, contained within it: the ventricles and the vascular bed. This compartment’s transmural pressure (${ICP}_{{BR}_{TM}}$) as a function of its volume is described by a polynomial (Eq. 2):

2 $${ICP}_{{BR}_{TM}}={a}_{b}{{V}_{BR}}^{3}+{b}_{b}{{V}_{BR}}^{2}+{c}_{b}{V}_{BR}+{d}_{b}$$

where ${V}_{BR}$ is the total volume enclosed by the pia, including the volume in the ventricles and in the blood vessels. Coefficient values ${a}_{b}$, ${b}_{b}$, ${c}_{b}$ and ${d}_{b}$ are detailed in Appendix.

Ventricular pressure is defined as follows: external pressure is the ${ICP}_{BR}$ and the ventricular transmural pressure $IC{P}_{{VEN}_{TM}}$ is defined as a function of its own volume as described by a polynomial (Eq. 3):

3 $$IC{P}_{{VEN}_{TM}}={a}_{v}{{V}_{VEN}}^{3}+{b}_{v}{{V}_{VEN}}^{2}+{c}_{v}{V}_{VEN}+{d}_{v}$$

where ${V}_{VEN}$ is the ventricular volume. Coefficient values ${a}_{v}$, ${b}_{v}$, ${c}_{v}$ and ${d}_{v}$ are detailed in Appendix.

The cerebrovascular circulation within the cranium is subdivided into three volume components: arterial and arteriolar, C_A_, capillary, C_C_ and venous, C_V_. The pressures in the three sub-compartments, arteriolar ${P}_{ARL}$, capillary ${P}_{C}$, and venous ${P}_{V}$ were described as a function of their blood volumes ${V}_{A}$, ${V}_{C}$, and ${V}_{V}$ respectively, by linear expressions (Eq. 4):

4 $${P}_{n}=\frac{{V}_{n}}{{C}_{n}}$$

As noted above, the superior sagittal sinus is the only intracranial compartment that maintains a pressure lower than ICP and is the site for both venous outflow and CSF reabsorption.

The spinal thecal sac is connected to the intracranial space through fluid conduit between the intracranial and spinal subarachnoid spaces. Pressure–volume relations in the sac were also described by a polynomial (Eq. 5):

5 $${P}_{SPI}={a}_{t}{{V}_{SPI}}^{2}+{b}_{t}{V}_{SPI}+{c}_{t}$$

where ${V}_{SPI}$ is the spinal thecal sac volume. Coefficient values ${a}_{t}$, ${b}_{t}$, and ${C}_{t}$ are detailed in Appendix.

Importantly, in the model transmural pressures of the ventricular and brain compartments are very small; however, they are required to maintain compartment volumes. For example, the ventricle is enclosed by the brain on its outer margin. The pressure in the ventricle (P_VEN_) is slightly greater than the pressure in the brain at baseline physiological conditions, thereby maintaining a positive transmural pressure and ventricular volume. When this normal physiological condition is reversed by pathophysiological processes, collapse of the compartment occurs.

Production rates of CSF (Q_CSF_) and ISF (Q_ISF_) in the model are constant (Table 1), in line with experimental evidence that supports a constant rate of production independent of changes in ICP [30]. Initial baseline CBF (Q_CBF_) is 910 ml/min, but varies in relation to changing volume and pressure in the other model compartments or changes in resistance within the cerebrovascular tree.

**Table 1:** Model baseline parameters

Component	Description	Value
${V}_{A}$	Arterial-arteriolar volume	20 [ml]
${V}_{C}$	Capillary volume	100 [ml]
${V}_{V}$	Venous volume	30 [ml]
${V}_{VEN}$	Ventricular volume	25 [ml]
${V}_{BR}$	Brain volume	1400 [ml]
${V}_{SAS}$	Subarachnoid space volume	35 [ml]
${V}_{SPI}$	Spinal thecal sac volume	70 [ml]
${V}_{TOT}$	Total cranial volume	1610 [ml]
${P}_{AO}$	Aortic pressure	93 [mmHg]
${P}_{ARL}$	Arteriolar pressure	64 [mmHg]
${P}_{C}$	Capillary pressure	30 [mmHg]
${P}_{V}$	Venous pressure	17 [mmHg]
${P}_{SSS}$	Superior sagittal sinus pressure	7.4 [mmHg]
${ICP}_{VEN}$	Ventricular pressure	10.8 [mmHg]
${ICP}_{BR}$	Brain intraparenchymal pressure	10.5 [mmHg]
${ICP}_{SAS}$	Subarachnoid space pressure	9.5 [mmHg]
${P}_{SPI}$	Spinal thecal sac pressure	9.5 [mmHg]
${Q}_{CSF}$	CSF production rate	0.0042 [ml${\text{ sec}}^{ - 1}$]
$Q_{ISF}$	Interstitial fluid production rate	0.00083 [ml${\text{ sec}}^{ - 1}$]
$Q_{CBF}$	Cerebral blood flow	910 [ml${\text{ min}}^{ - 1}$]
$R_{A}$	Arterial-arteriolar resistance to flow	1.9 [mmHg s${\text{ ml}}^{ - 1}$]
$R_{C}$	Capillary resistance to flow	2.5 [mmHg s${\text{ ml}}^{ - 1}$]
$R_{V}$	Venous resistance to flow	0.8 [mmHg s${\text{ ml}}^{ - 1}$]
$R_{BV}$	Bridging veins resistance to flow	0.7 [mmHg s${\text{ ml}}^{ - 1}$]
$R_{BBB}$	Brain Blood Barrier	10,000,000 [mmHg s${\text{ ml}}^{ - 1}$]
$R_{VEN}$	Ventricular resistance to CSF flow	250 [mmHg s${\text{ ml}}^{ - 1}$]
$R_{BF}$	Resistance to bulk flow of cerebral interstitial flow	1200 [mmHg s${\text{ ml}}^{ - 1}$]
$R_{OUT}$	Resistance to CSF reabsorption and bulk flow to the subarachnoid space	280 [mmHg s${\text{ ml}}^{ - 1}$]
$R_{FM}$	Resistance to CSF flow through the Foramen magnum	15 [mmHg s ${\text{ml}}^{ - 1}$]
$C_{A}$	Arterial-arteriolar compliance	0.4 [ml${\text{ mmHg}}^{ - 1}$]
$C_{C}$	Capillary compliance	5 [ml${\text{ mmHg}}^{ - 1}$]
$C_{V}$	Venous compliance	5 [ml${\text{ mmHg}}^{ - 1}$]
$C_{VEN}$	Ventricular compliance	see Eq. 3
$C_{BR}$	Brain compliance	see Eq. 2
$C_{SAS}$	Subarachnoid space compliance	see Eq. 1
$C_{SPI}$	Spinal thecal sac compliance	see Eq. 5

Fluid conduits connect between some of the different compartments. In the model, resistors represent viscous resistance to fluid flows within the brain and ventricular system and through the cerebrovascular tree. In the cerebrovascular circulation, R_A_, R_C_, and R_V_ and represent viscous resistance to flow in the circulation. The resistors in the vascular bed are affected by the transmural pressure of the vascular wall that is exposed to ICP_BR_. Changes in ICP_BR_ affect the volume inside the vessel that in-turn alters the resistance. Radius changes affects resistance, according to Hagen-Poiseuille's law for a cylinder (Eq. 6):

6 $$R=\frac{8\mu L}{\pi {r}^{4}}=\frac{{R}_{0}}{{\left(\frac{V}{{V}_{0}}\right)}^{2}}$$

$$where V=\pi {r}^{2}L$$

$\mu$ is viscosity, L segment length and r is the radius. ${R}_{0}$ is initial resistance of the compartment with compartment volume $V={V}_{0}$ at baseline conditions.

The normally very high resistance to the passage of fluid from the cerebrovascular circulation into the brain parenchyma is depicted by R_BBB_ which has a very high value under normal physiological conditions. The model depicts pathophysiological disturbance that impair the normally nearly impenetrable blood–brain barrier as a decreasing R_BBB._

The resistances to flow of CSF and ISF are depicted as follow: R_VEN_ represents resistance to flow within the ventricles and out of the ventricular system into the subarachnoid space. R_BF_ represents resistance to the bulk flow of cerebral ISF within the brain’s extracellular space and out to the subarachnoid space where it merges with the circulating CSF. R_OUT_ represents the resistance to CSF absorption into the superior sagittal sinus. Lastly, R_FM_ represents the resistance to flow of CSF from the intracranial subarachnoid compartment to the spinal subarachnoid compartment. The resistors to the flow of CSF and ISF are affected by the volume of CSF or ISF in a compartment through which they flow. For example, an extracellular compartment with large fluid content will result in larger space through which cerebral ISF can flow leading to a lower resistance (low R_BF_). In contrast, when the volume of the extracellular space is low as occurs in brain swelling, the resulting denser cellular space leads to greater resistance to cerebral ISF flow through the constricted ECF channels (high R_BF_).

The solution of the model involves solving seven differential equations. Due to the nonlinear behavior of the parameters’ values and their dependency on pressure and volume values, the Euler method was employed to solve the model and reach a steady state solution for each pathological condition. The Euler method was used as follows:

Volume update for every capacitor:

$${V}_{n}\left(i\right)={V}_{n}\left(i-1\right)+\left({Q}_{n}\left(i-1\right)-{Q}_{n+1}\left(i-1\right)\right)\Delta t$$

where n is capacitor index and $i$ is discrete time.

Pressure update:

$${P}_{n}\left(i\right)={f}_{n}({V}_{n}\left(i\right))$$

where the function is one of those described above (exponential or polynomial)

Parameter values update:

$${R}_{n}\left(i\right)=\frac{{R}_{0n}}{{\left(\frac{{V}_{n}(i)}{{V}_{0n}}\right)}^{2}}$$

Flow calculation:

$${Q}_{n}\left(i\right)=\frac{{P}_{n}\left(i\right)-{P}_{n+1}(i)}{{R}_{n}(i)}$$

Table 1 details initial baseline values and the model components’ characteristic pressure and volume relations. Most baseline values were designated based on normal values obtained from the literature, while others whose values may be uncertain due to the paucity of data in the literature were derived and estimated on the basis of normal physiological pressures and flows.

### Simulation of physiological perturbations

Selected physiological perturbations were assessed to test model validity and to assess whether model simulations accorded with expected cerebral physiological responses. We simulated rapid intracranial injection of increasing volumes of fluid at normal ICP in order to establish the pressure–volume response to a rapid change in intracranial volume. In addition, we simulated hyperventilation and hypoventilation by altering the arterial-arteriolar resistance (${R}_{A}$) in the cerebrovascular component of the model, since this is the primary mechanism by which ventilation changes affect CBF. We assessed the model’s ability to appropriately simulate the changes in cerebral physiological parameters that are induced by different pathophysiological phenomena that lead to raised ICP, including hydrocephalus (obstructive and absorptive) and cerebral edema induced by either cellular swelling or blood–brain barrier breakdown. Ventricular obstruction was modelled by an increasing resistance to flow in the ventricles (${R}_{VEN}$), absorptive hydrocephalus was modelled by increasing an increasing resistance to CSF reabsorption (${R}_{OUT}$), cellular swelling with an increasing resistance to the flow of cerebral interstitial fluid was modelled by increasing the resistance to the bulk flow of cerebral interstitial fluid (${R}_{BF}$), while a breakdown in the blood–brain barrier was simulated by a decrease in the resistance to flow across the BBB (${R}_{BBB}$). Changes in volume distributions across the different intracranial compartments (brain tissue, ventricles, subarachnoid space, and the vascular compartments) induced by these alterations once the model reached steady-state were determined. The changes in the ICP waveform with rising ICP were also investigated. MATLAB (The MathWorks Inc., Natick, Massachusetts) was used for intracranial fluid dynamics model simulations.

## Results

**Fig. 2:**
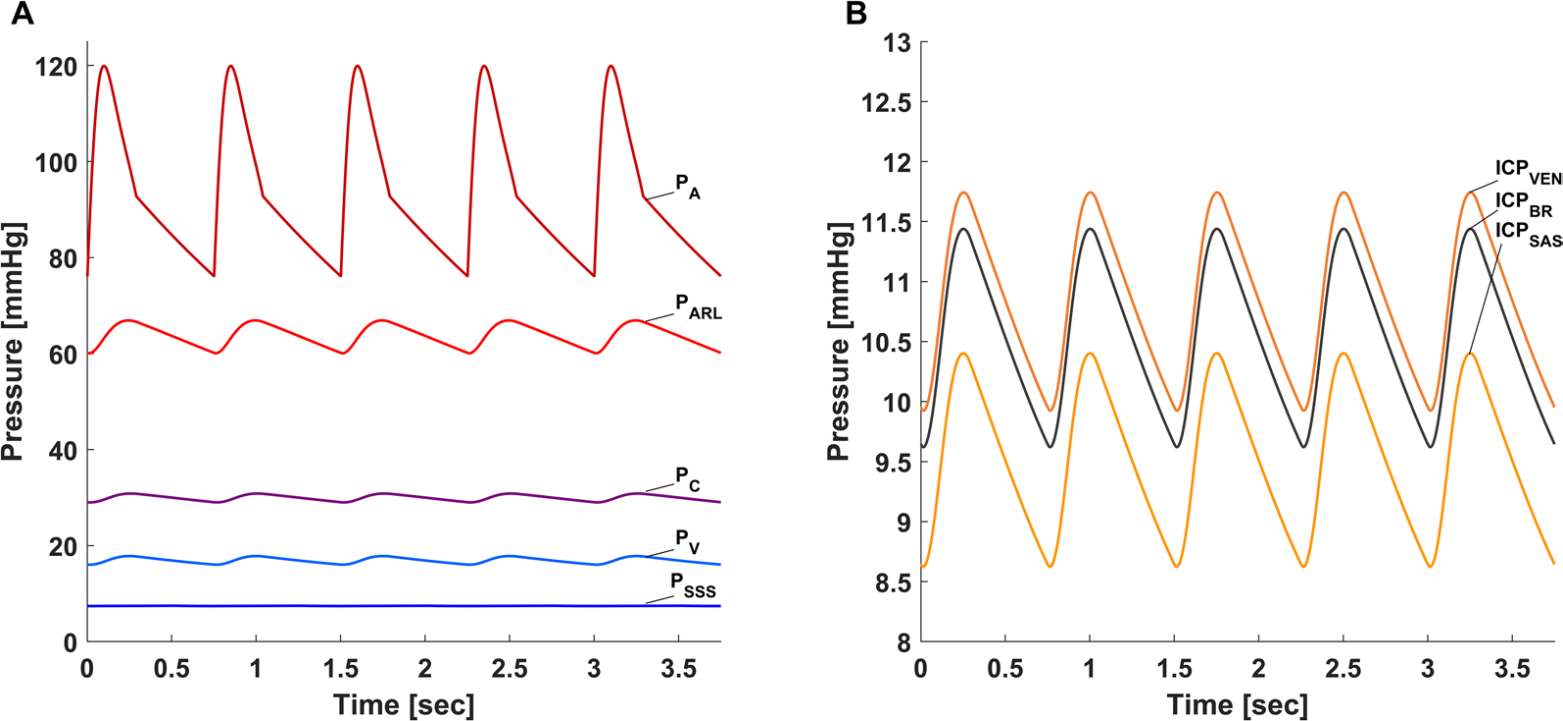
**A** Waveform and hierarchies of pressures in the cerebral vasculature at baseline ICP. Systemic arterial pressure (P_A_) with systolic peaks around 120 mmHg is followed by cerebral arteriolar pressure (P_ARL_) which is somewhat lower. Pressures in the capillary bed (P_C_) and veins (P_V_) is markedly lower and displays a flattened waveform typical of capillary and venous circulation. Pressure in the sagittal sinus (P_SSS_) is the lowest in the intracranial space and is typically lower than ICP. **B** Waveform and hierarchies of pressures in the ventricles (ICP_VEN_), brain (ICP_BR_), and subarachnoid space (ICP_SAS_). At baseline, ventricular pressure is slightly higher than brain intraparenchymal pressure preventing ventricular collapse. Subarachnoid pressure (ICP_SAS_) is slightly lower than both ventricular and brain intraparenchymal pressure, allowing flow of both ventricular CSF and cerebral interstitial fluid into the subarachnoid space

**Fig. 3:**
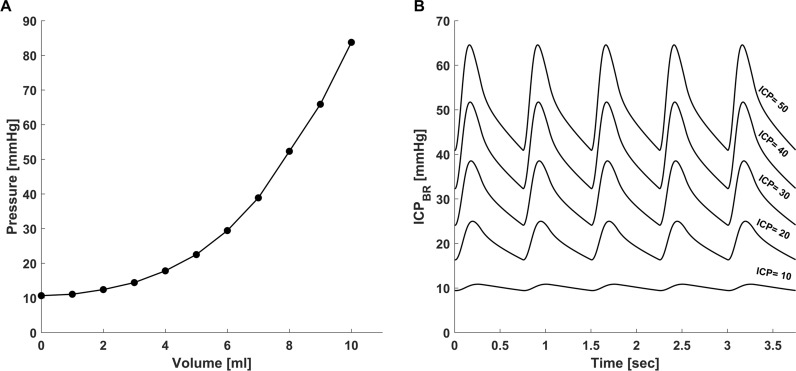
**A** Pressure–volume curve obtained by simulating injection of fluid of increasing volume into the intracranial space demonstrating the expected exponential-like rise in ICP with higher volumes. **B** ICP waveforms with increasing ICP. As brain parenchymal ICP (ICP_BR_) rises from 10 to 50 mmHg, the model demonstrates the typical increase in ICP pulse pressure that results from decreased intracranial compliance

**Fig. 4:**
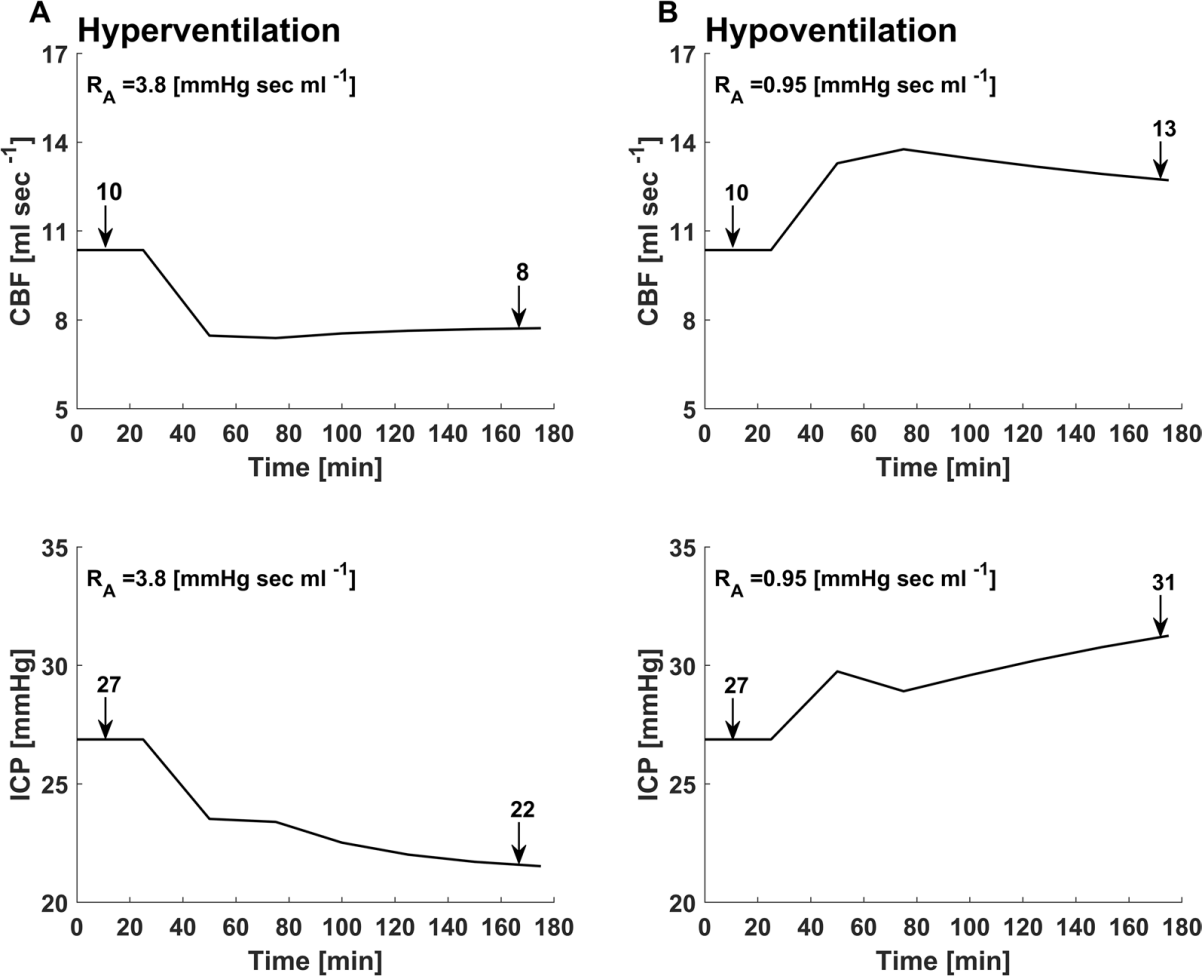
**A** Cerebral blood flow (CBF) and ICP changes seen with hyperventilation. As arteriolar resistance (R_A_) increases, both CBF and ICP decrease in conjunction with each other. **B** Conversely, a decreased arteriolar resistance results in increased CBF and ICP, in accordance with expected physiological norms

Initial assessment of the model demonstrated stable values for pressure, volume, and flow in all three model elements of brain, CSF, and blood. The hierarchy of pressures between the ventricles, cerebral parenchyma, and subarachnoid space on the one hand, and the cerebral venous compartment on the other, was maintained in the expected manner (Fig. 2). Importantly, simulations of rapid intracranial volume injections, demonstrated the exponential-like intracranial pressure–volume curve (Fig. 3A). Rising ICP_BR_ also resulted in changes to the ICP waveform that are typically seen when ICP is elevated, including a progressively increasing ICP pulse amplitude typical to states of high intracranial elastance (Fig. 3B). Changes in arteriolar resistance led to the expected changes in CBF and ICP_BR_, simulating the decrease in ICP induced by hyperventilation and the increase in ICP induced by hypoventilation (Fig. 4). Hyperventilation resulted in a 20% decrease in CBF from baseline and an 18% decrease in ICP. Conversely, hypoventilation resulted in a 30% increase in CBF from baseline and a 15% increase in ICP.

**Fig. 5:**
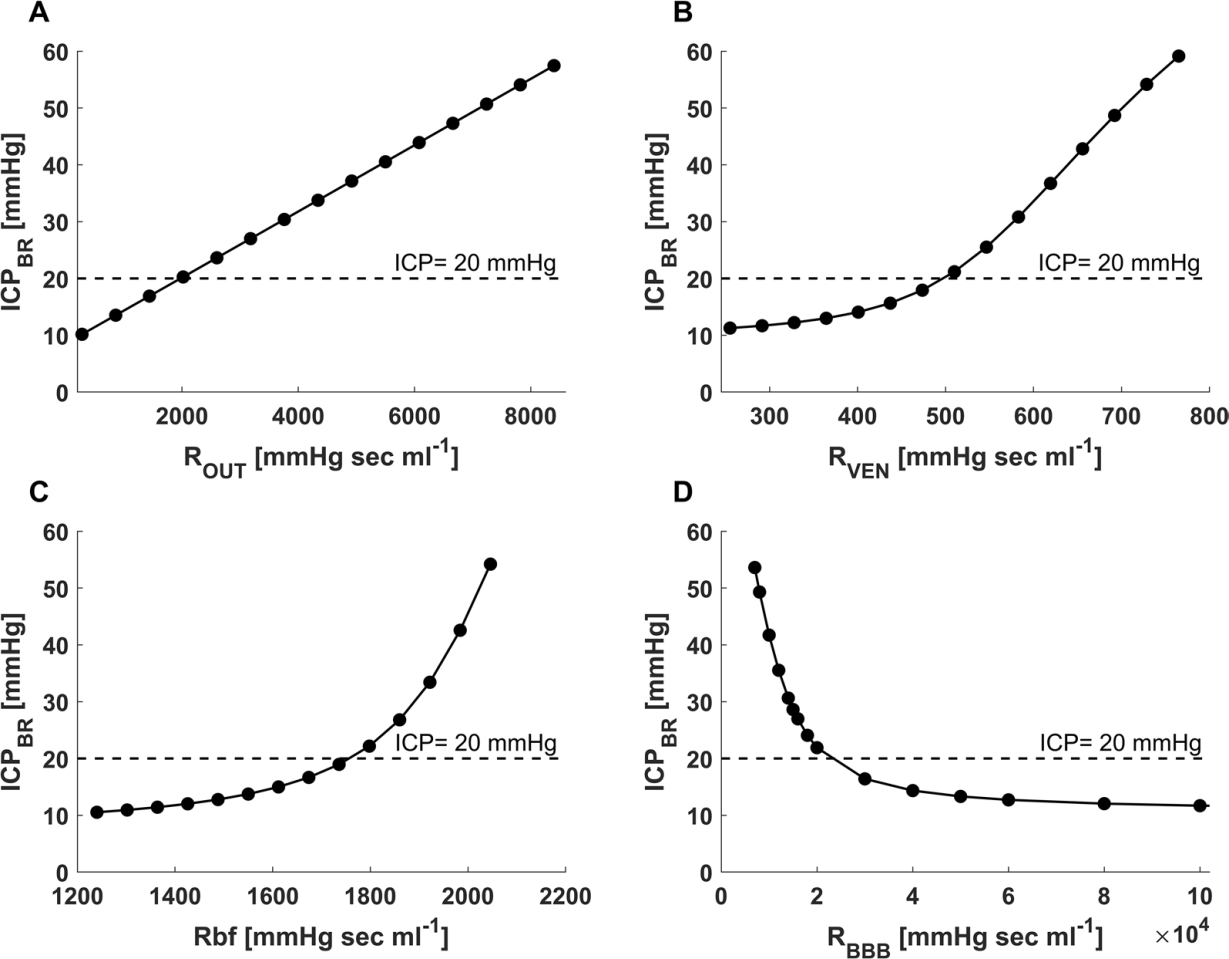
Changes in ICP with changes in resistances in the model. **A** Increased resistance to CSF outflow (R_OUT_) that occurs with non-absorptive hydrocephalus leads to the expected rise in brain intraparenchymal pressure (ICP_BR_). **B** Increased resistance to CSF flow in the ventricular system (R_VEN_) which is typically seen in obstructive hydrocephalus also leads to a sharp rise in ICP_BR_. **C** A rise in resistance to the bulk flow of cerebral interstitial fluid (R_BF_) leads to a pronounced rise in ICP_BR_ which is characteristic of cerebral edema. **D** Similarly, a disruption of the blood–brain barrier (R_BBB_) leads to a marked rise in ICP_BR_ as the R_BBB_ decreases and allows more fluid to cross from the cerebrovascular compartment into the brain

As expected, the lumped parameter model demonstrated rising intracranial pressure under conditions of obstructive hydrocephalus, absorptive hydrocephalus, and cerebral edema (Fig. 5A–D). While ICP rises in each of these cases, the effect on the volumes in the different intracranial compartments varies between simulations of different pathophysiological conditions. When non-absorptive hydrocephalus (high R_OUT_) or obstructive hydrocephalus (high R_VEN_) is simulated, ventricular volume increases substantially to 140% and 152% of baseline values, respectively, as ICP reaches 20 mmHg. In contrast, when cerebral edema is simulated by an increased R_BF_ or decreased R_BBB_, ventricular volume decreases markedly to 8% and 12% of baseline values, respectively, as ICP reaches 20 mmHg.

**Fig. 6:**
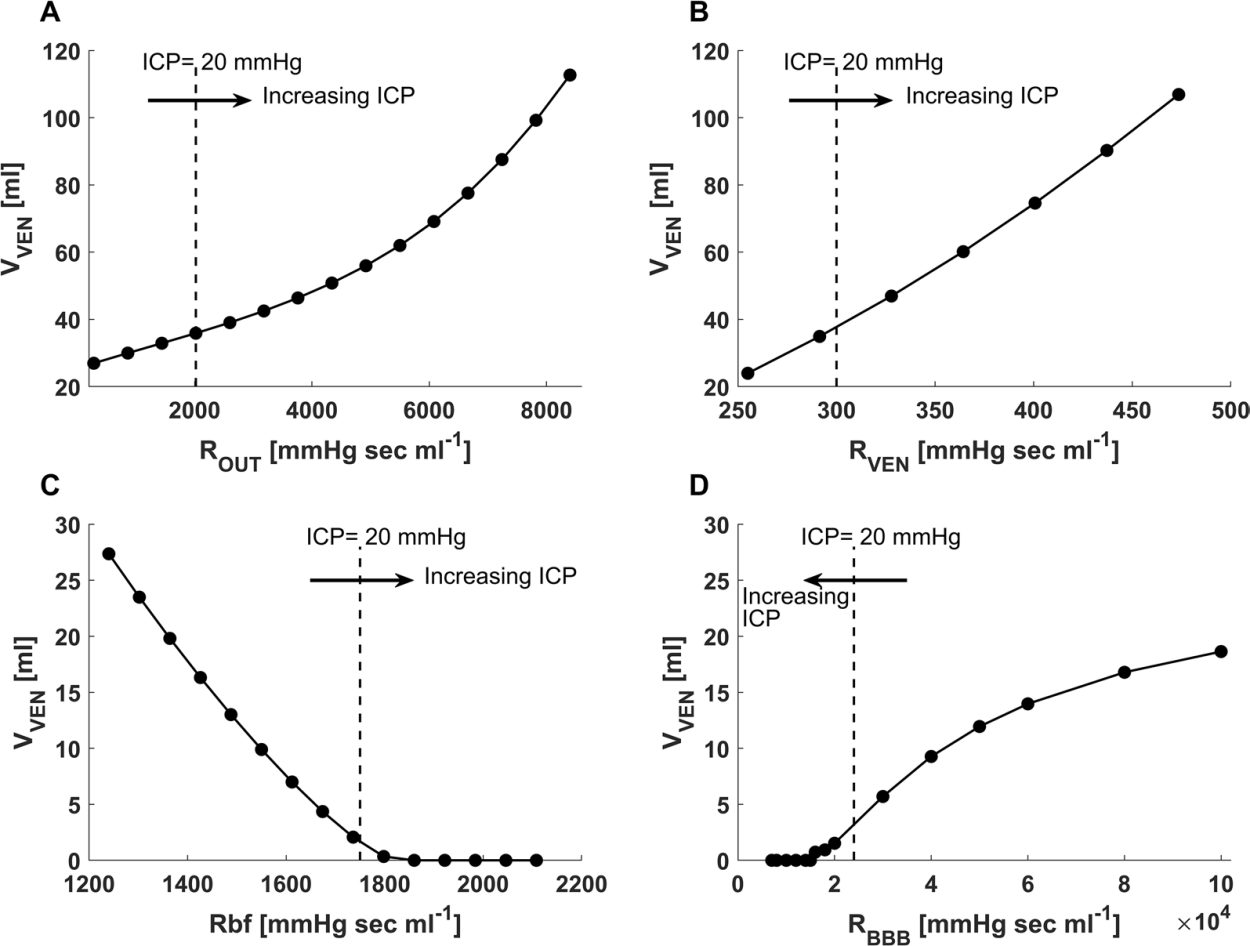
Ventricular volume as a function of changes in resistances in the model. **A** As expected, increased CSF outflow resistance (R_OUT_) representing non-absorptive hydrocephalus leads to increased ventricular volume (V_VEN_). **B** Increased resistance to CSF flow in the ventricular system (R_VEN_) which depicts obstructive hydrocephalus also leads to increased V_VEN_. **C** Conversely, an increase in the resistance to bulk flow of cerebral interstitial fluid (R_BF_) that occurs with cerebral edema results in a decrease and then collapse of V_VEN_, simulating the clinical situation in which ventricular volume decreases markedly as the brain swells. **D** A decreasing resistance to movement of fluid across the blood–brain barrier (R_BBB_) leads to a rapid decrease in V_VEN_, simulating the situation in which blood–brain barrier disruption leads to brain swelling with a concomitant decrease in ventricular volume

Table 2 details the changes in model output parameters that are induced by a change in the input values of R_OUT_, R_VEN_, R_BF_, and R_BBB_ which lead to an ICP rise to a level of 30 mmHg. The changes in the volumes of the brain, ventricles, and subarachnoid space compartments are detailed in the Table. Importantly, in the model CBF decreases substantially with the rise in ICP. The results demonstrate that model output corresponds to accepted patterns associated with each of the distinct pathophysiological conditions. The model clearly indicates that simulating cerebral edema with an increase in the resistance to the flow of cerebral interstitial fluid (${R}_{BF}$) leads not only to raised ICP_BR_ (Fig. 5C), but also to an increase in total brain volume, and a marked decrease in ventricular and subarachnoid space volume (Table 2), accurately replicating the physiological changes known to occur with brain edema. We also modeled the effect of changes in the integrity of the blood–brain barrier on cerebral physiological parameters (Table 2). Not surprisingly, a breakdown of blood–brain barrier integrity represented by a decrease in ${R}_{BBB}$, resulted in an increase in the volume of the brain component in the model (Table 2) leading to a rise in ICP_BR_ (Fig. 5D) and a marked decrease in ventricular volume (Fig. 6D) that contrasts with the increasing ventricular volume that occurs with non-absorptive or obstructive hydrocephalus (Fig. 6A, B). ICP_BR_ increased dramatically as R_BBB_ decreased to low levels indicating an exponential-like effect of a rising permeability to fluid shifts across the BBB (Fig. 5D).

**Table 2:** Changes in model output parameters as ICP rises from baseline to 30 mmHg under four conditions

	R_OUT_	R_VEN_	R_BF_	R_BBB_
Total brain volume ($ml$)	+ 16	− 62	+ 94	+ 94
Ventricular volume ($ml$)	+ 20	+ 133	− 25	− 25
Subarachnoid space volume ($ml$)	+ 10	− 25	− 24	− 24
Cerebral blood volume ($ml$)	− 41	− 41	− 40	− 40
CBF (${ml 100 gr}^{-1}$ ${min}^{-1}$)	− 26	− 26	− 26	− 26

The Table describes the output parameters under four input conditions: increased resistance to CSF absorption, increased resistance to ventricular CSF flow, increased resistance to bulk flow of ISF, and breakdown of the BBB. R_OUT_: resistance to cerebrospinal fluid (CSF) outflow; R_VEN_: resistance to CSF flow in the ventricular system; R_BF_: resistance to bulk flow of cerebral interstitial fluid; R_BBB_: resistance to flow of fluid across the blood–brain barrier; CBF: cerebral blood flow

## Discussion

The four-compartment model we describe simulates the important interactions of brain, blood, and CSF within the closed cranium and accurately replicates important and well-accepted aspects of intracranial physiology. The model replicates the pressure–volume curve in response to rapid volume injection into the intracranial space (Fig. 3) and the responses to hyper and hypoventilation (Fig. 4). Importantly, the incorporation of a novel compartment to the model, the brain, through which flows cerebral interstitial fluid, adds an essential component that has not been previously included in models of intracranial hydrodynamics. This is in effect a modification of Davson’s original formulation:

7 $${ICP}^{D}={Q}_{ CSF}^{D}\times {R}_{ OUT}^{D}+{P}_{SSS}$$

where the superscript, ^D^, denotes the parameters referred to by Davson: ICP^D^ a lumped ICP for a single intracranial compartment, Q^D^_CSF_ representing flow of all CSF within the cranium, which in the context of the current model corresponds to flow of CSF in the subarachnoid space or the sum of ISF and CSF flow, and R^D^_OUT_ representing the resistance to reabsorption of CSF into the superior sagittal sinus. Rather than treating the intracranial space as a single lumped compartment with a unitary pressure, the model we describe stresses the small but important differences between pressures in each of the different cerebral compartments, ventricles, brain, and subarachnoid space. In our model, steady-state pressure in the brain parenchyma and subarachnoid space, respectively, may be described as follows:

8 $${ICP}_{BR}={Q}_{ISF}\times {R}_{BF}+{ICP}_{SAS}$$

9 $${ICP}_{SAS}=\left({Q}_{ISF}+{Q}_{CSF}\right)\times {R}_{OUT}+{P}_{SSS}$$

Substituting Eq. 9 in Eq. 8 yields Eq. 10.

10 $${ICP}_{BR}={Q}_{ISF}\times {R}_{BF}+\left({Q}_{ISF}+{Q}_{CSF}\right)\times {R}_{OUT}+{P}_{SSS}$$

This modification of the Davson equation considers both CSF and ISF production and flow as essential elements in determining steady-state cerebral intraparenchymal pressure. This additional element is vital because it allows modelling of pathologies in which cerebral edema is the primary component leading to raised ICP and an increased resistance to bulk flow of ISF. Our results clearly demonstrate the rise in ICP caused by an increase in R_BF_ (Fig. 5) and the consequent changes in volumes of the other intracranial compartments that occur with brain swelling (Table 2). Pathologies in which cerebral edema predominates, either as the primary insult or the consequence of secondary brain injury occur frequently in neurocritical care and continue to present difficult therapeutic challenges. The current model offers the ability to separate two distinct pathways leading to ICP elevation, those caused by obstruction to CSF flow (${R}_{VEN}$, ${R}_{OUT}$) and those caused by swelling of the brain itself which leads to an increased resistance to cerebral interstitial fluid flow (${R}_{BF}$). The model also depicts the changes caused by a breakdown of the normally nearly impenetrable blood–brain barrier (${R}_{BBB}$) (Figs. 5D, 6D). The ability to model these important pathophysiological conditions may provide potential benefit by affording an improved categorization of the underlying pathophysiology that leads to raised ICP. Crucially, in addition to demonstrating raised ICP, the model replicates well known clinical manifestations that occur with brain swelling such as a marked decrease in volume of the ventricles and the subarachnoid space (analogous to cisternal and sulcal effacement) which most previous models of intracranial hydrodynamics have not addressed (Table 2). Opening of the blood–brain barrier is another well-recognized pathological process known to lead to severe cerebral edema and raised ICP [31–33]. The current model is successful in emulating the multifaceted effects of increased BBB permeability, indicating its potential utility in modelling the blood–brain barrier disruption that occurs with brain injury. Importantly, the model simulates the often-complex multivariate interactions between key cerebral physiological parameters. By simulating a primary disturbance in one of four key resistances (${R}_{VEN}$, ${R}_{OUT}$, ${R}_{BF}$, and ${R}_{BBB}$) the model is able to demonstrate the ensuing changes in the volumes, pressures, and resistances in each of the intracranial compartments. The model also depicts the changes in intracranial compliance that occur with rising ICP and with a rapid volume load to the intracranial space (Fig. 3A). Similar to previous models of ICP pressure dynamics it replicates the changes in the ICP waveform that result from raised ICP and decreased intracranial compliance (Fig. 3B) [11, 34]. The ability to study interrelationships is an important advantage of lumped parameter modelling. Although this is a preliminary study to establish the theoretical underpinnings of modelling intracranial pressure–volume dynamics in relation to the resistance to cerebral ISF flow, our hope is that future translational studies will be able to build on this work and use large animal experimental models or data from brain injured patients in order to assess the model’s ability to help predict thresholds of intracranial volume-reserve capacity in cerebral edema.

Previous models of intracranial hydrodynamics and cerebrovascular circulation established the underlying principles needed to depict the interactions between the pressure, volume, and flow of CSF and CBF [35]. However, a substantial gap in these models remained in that none described the important changes that occur when the brain itself swells and the flow of cerebral interstitial fluid is impaired by that swelling. This lacunae in the models of intracranial hydrodynamics is of crucial importance because many of the pathologies dealt with by neurosurgeons and neurocritical care specialists involve severe cerebral edema. Without a means to accurately represent the changes that occur when the brain swells a substantial gap exists in the ability of models of intracranial physiology to describe the interactions between cerebral physiological parameters in a clinically meaningful way. The physiological importance of the flow of cerebral interstitial fluid has received renewed attention in recent years [36–38]. When cerebral intracellular swelling occurs the normal flow of brain ISF may be impaired [39, 40] with important consequences for ICP. Intracellular swelling leads to a substantial reduction in the volume of the cerebral extracellular space [41–43], leaving smaller constricted channels through which the cerebral ISF must flow. Under these conditions, the flow of cerebral ISF is impaired by cellular swelling. The model depicts this pathophysiological condition as an increased resistance to bulk flow of ISF through the brain parenchyma (increased R_BF_). An increasing R_BF_ value represents the heightened resistance to the efflux of the water that is a by-product of cerebral metabolism out of the brain parenchyma by way of its normal passage from the extracellular space into the subarachnoid space. The effects of the resulting increased resistance to cerebral ISF flow are apparent in the model where a marked rise in ICP results from an increase in ${R}_{BF}$. The resulting rise in ICP may, in turn, further affect other cerebral physiological parameters leading to a cascade effect with deleterious consequences. An advantage of the lumped parameter model is that it allows the simulation of these interactions.

The model we describe also needs to be examined in light of recent controversies regarding cerebral ISF production and flow. In recent years, the development of the glymphatic and intramural peri-arterial drainage (IPAD) theories has challenged traditional conceptions of ISF flow within the brain [44–48]. Strongly held views are held by both sides of the on-going debate regarding the experimental evidence and whether it supports the glymphatic theory or more traditional theories of ISF production and circulation. We sought to describe a model that describes the importance of ISF circulation as a key component in determining ICP, especially under conditions of brain edema. We aimed to ensure that the model is compatible with both traditional and glymphatic theories of cerebral ISF circulation. The model only assumes that ISF is produced at a constant rate (0.00083 ml/sec) as noted in Table 1. This comprises just under 20% of the summed total of CSF plus ISF production, which is in line with ratio of production of metabolic water produced by cellular metabolism in relation to the rate of CSF production. Whether this fluid is produced by mechanisms proposed by the glymphatic theory, the IPAD theory, or by traditional theories of ISF production and flow does not bear directly on the model’s results. The model postulates that cerebral ISF flows from the extracellular space of the brain parenchyma into the subarachnoid space. An important aspect of the model is that it obeys the principles of hydrodynamics in that fluid flows from a higher pressure into a compartment of lower pressure. The subarachnoid space which envelopes the brain is postulated to have a slightly lower pressure than both the brain parenchyma which it surrounds and the ventricles which empty directly into it. The slightly lower pressure of the intracranial subarachnoid space results from the fact that it itself is the site of CSF exit out of the cranium by way of reabsorption into the superior sagittal sinus. Importantly, when cerebral swelling or hydrocephalus lead to a smaller subarachnoid space, resistance to flow within this compartment increases and CSF reabsorption is impaired. While it is difficult to definitively establish whether small pressure differences between the intracranial compartments exist, it is not unreasonable to postulate that they may occur. The small number of clinical studies that have measured ICP concomitantly in the ventricles and brain parenchyma have found differences in these simultaneous measurements [49–53]. A meta-analysis of these studies identified that 70% of concomitant measurements in the same patient could vary by as much as ± 6 mmHg and an overall mean pressure difference of 1.6 mmHg exists [54]. Admittedly, it remains difficult to ascertain to what degree these differences are due to actual differences in pressure between compartments and to what degree they may be due to measurement error. Further meticulous experimental and clinical studies will be required to establish whether the small differences in transmural pressures proposed in the model can be demonstrated and quantified by experimental data.

### Limitations

Our study has several limitations. This is a preliminary work that seeks to add the additional element of cerebral interstitial fluid flow to the previous models that described ICP in terms of CSF hydrodynamics and the cerebral vascular circulation alone. In order to study the important first-order effects of adding the novel element of cerebral interstitial fluid flow, we did not incorporate a dynamic model of CBF with autoregulation into the current version of the model. This will be an important goal of future studies that seek to investigate the important interplay between cerebral edema, raised ICP, and the compensatory ability to maintain adequate cerebral perfusion. Second, the current model does not inherently separate the cerebral intracellular and the cerebral extracellular compartments. Further modifications to the model may achieve an improved description of different pathophysiological conditions if this separation is incorporated. Another potential limitation of this study is that some physiological parameters incorporated into the model, such as resistance to flow of CSF in the ventricles and resistance to cerebral ISF flow, are difficult to measure in clinical practice. The estimation of these parameters, which relies on derivations from more easily measured pressures, volumes, and flows in the intracranial system, introduces uncertainty into the model. Though unlikely to alter model behavior, the exact degree to which these uncertainties may modify model output will require further investigation. Lastly, the clinical utility of the model can only be truly assessed with translational studies that incorporate the collection of cerebral physiological parameters from either a large animal model or brain injured patients that are “plugged into the model” in order to assess its ability to predict thresholds of intracranial volume-reserve capacity. Although we did not have the resources to perform these resource-intensive studies, our hope is that future investigations will aim to determine whether the model may be a useful platform to achieve this goal.

## Conclusions

The four-compartment model of intracranial physiology incorporates a depiction of cerebral interstitial fluid flow and resistance that allows modelling of brain edema. The model accounts for the complex interactions that occur between different cerebral physiological parameters and accurately depicts the changes in pressure, volume, and resistances to flow in the different intracranial compartments under specific pathophysiological conditions. It may serve as a platform for improved modelling of the cerebral edema and blood–brain barrier disruption that occur following brain injury.

## Data Availability

The datasets used and/or analyzed during the current study are available from the corresponding author on reasonable request.

## Appendix

Equation A1 presents the ventricular steady state pressure:

A1 $${ICP}_{VEN}={Q}_{CSF}\times {R}_{VEN}+{ICP}_{SAS}$$

where ${Q}_{CSF}$ is CSF flow, ${R}_{VEN}$ is ventricular resistance to flow and ${ICP}_{SAS}$ is subarachnoid pressure.

Ventricular resistance to flow (${R}_{VEN}$) presented as an ICP-dependent nonlinear function:

A2 $${R}_{VEN}={R}_{{VEN}_{0}}+10\times {e}^{(0.048\times {ICP}_{BR})}$$

where ${R}_{{VEN}_{0}}$ is a constant set to 250 [mmHg sec ${\text{ml}}^{ - 1}$] and ${ICP}_{BR}$ is brain intraparenchymal pressure.

Values for each of the coefficients in the polynomial equations appearing in the Methods section are as follows:

Coefficient values for Eq. 1, subarachnoid space pressure:

$${a}_{s}=2.963$$

$${b}_{s}=0.4664$$

$${c}_{s}=7.5$$

Coefficient values for Eq. 2, brain parenchymal pressure (transmural):

$${a}_{b}={2\times 10}^{-7}$$

$${b}_{b}=-{3\times 10}^{-4}$$

$${c}_{b}=0.1637$$

$${d}_{b}=-32.342$$

Coefficient values for Eq. 3, ventricular pressure (transmural):

$${a}_{v}={3\times 10}^{-7}$$

$${b}_{v}=-{1\times 10}^{-4}$$

$${c}_{v}=0.0181$$

$${d}_{v}=-0.1159$$

Coefficient values for Eq. 5, spinal thecal sac pressure:

$${a}_{t}={2\times 10}^{-4}$$

$${b}_{t}=-{3\times 10}^{-4}$$

$${C}_{t}=7.0386$$

## Abbreviations

$${P}_{A}$$: Arterial pressure
$${P}_{ARL}$$: Arteriolar pressure
$${P}_{C}$$: Capillary pressure
$${P}_{V}$$: Venous pressure
$${ICP}_{VEN}$$: Ventricular pressure
${ICP}_{BR}$: Brain intraparenchymal pressure
${ICP}_{SAS}$: Subarachnoid space pressure
${P}_{SPI}$: Spinal thecal pressure
${P}_{SSS}$: Superior sagittal sinus pressure
${C}_{A}$: Arterial-arteriolar compliance
$${C}_{C}$$: Capillary compliance
$${C}_{V}$$: Venous compliance
$${C}_{VEN}$$: Ventricular compliance
$${C}_{BR}$$: Brain compliance
${C}_{SAS}$: Subarachnoid space compliance
${C}_{SPI}$: Spinal thecal sac compliance
${R}_{A}$: Arterial-arteriolar resistance to flow
${R}_{C}$: Capillary resistance to flow
${R}_{V}$: Venous resistance to flow
${R}_{BV}$: Bridging veins resistance to flow
${R}_{BBB}$: Brain Blood Barrier
${R}_{VEN}$: Resistance to CSF flow in the ventricles
${R}_{BF}$: Resistance to bulk flow of cerebral interstitial flow
${R}_{OUT}$: Resistance to CSF reabsorption and to bulk flow in the subarachnoid space
${R}_{FM}$: Resistance to CSF flow through the foramen magnum
$${V}_{A}$$: Arteriolar volume
$${V}_{C}$$: Capillary volume
$${V}_{V}$$: Venous volume
$${V}_{VEN}$$: Ventricular volume
$${V}_{BR}$$: Brain volume
${V}_{SAS}$: Subarachnoid space volume
${V}_{TOT}$: Total intracranial volume
${V}_{SPI}$: Spinal thecal sac volume
${Q}_{CSF}$: CSF production rate
${Q}_{ISF}$: Cerebral interstitial fluid production rate
${Q}_{CBF}$: Cerebral blood flow
